# Predictive model for bleeding after gastric submucosal dissection before and after guidelines: A single‐center retrospective study

**DOI:** 10.1002/deo2.153

**Published:** 2022-07-12

**Authors:** Keita Saito, Hironobu Nagumo, Takashi Ashikawa, Tomoyuki Funato, So Nakaji, Hiroki Matsui

**Affiliations:** ^1^ Department of Gastroenterology Kameda General Hospital Japan; ^2^ Department of Public Health Medicine, Graduate School of Medicine University of Tokyo Tokyo Japan

**Keywords:** anticoagulants, antithrombotic agents, bleeding, endoscopic submucosal dissection, risk factors

## Abstract

**Objectives:**

In July 2017, supplementary guidelines on anticoagulants, including direct oral anticoagulants, were published in Japan. We investigated the changes in endoscopic submucosal dissection (ESD) of gastric mucosal lesions after the publication of the supplement, examined the risk factors, and developed a predictive model for post‐ESD bleeding.

**Methods:**

We included 2272 gastric ESD cases from our hospital between May 2003 and June 2021 and classified them into two groups: 1789 cases before and 483 after the publication of the supplementary guidelines. A predictive model for post‐ESD bleeding was developed using the pre‐publication cohort data.

**Results:**

The proportion of patients receiving warfarin decreased (5.0% vs. 1.4%) and those receiving direct oral anticoagulants increased (1.2% vs. 6.8%) after the publication of the supplementary guidelines. Post‐ESD bleeding occurred in 61 patients, but there was no significant difference in the bleeding rate between the groups (50 [2.8%] vs. 11 [2.3%] patients, respectively). Five risk factors (number of antithrombotic agents, dialysis, heparin replacement, resection specimen size, and procedure time) were identified for model development. The C‐statistic for the model and post‐publication cohorts were 0.83 and 0.72, respectively. In the model, each risk factor for postoperative bleeding was scored, and the risk was classified into three levels according to the total score. Bleeding rates at low, intermediate, and high risks were 1.6%, 10.3%, and 38.9%, respectively.

**Conclusion:**

Despite changes in patient characteristics and clinical practice regarding ESD before and after the publication of the supplementary guidelines, we could still develop a simple and useful predictive model.

## INTRODUCTION

Endoscopic submucosal dissection (ESD) is commonly performed for gastric tumors,^1^, ^2^, ^3^ as it facilitates en bloc resection of the tumor and is less invasive than surgical resection.^4^, ^5^ Although infrequent, post‐ESD bleeding plagues endoscopists.^6^, ^7^, ^8^ Its risk factors include antithrombotic agents, such as warfarin, direct oral anticoagulants (DOACs), aspirin, and P2Y12 receptor antagonists.^9^, ^10^ In 2012, the Japan Gastroenterological Endoscopy Society published the “Guidelines for gastroenterological endoscopy in patients undergoing antithrombotic treatment.”^11^ In July 2017, it published a supplement^12^ specifying that DOACs should only be discontinued briefly when performing endoscopic procedures with a high risk for bleeding. Due to calls to verify the validity of these guidelines, we examined changes in ESD for gastric tumors after the publication of the supplement in a local community hospital. We also examined the risk factors for post‐ESD bleeding and developed a predictive model. Although predictive models have been constructed in previous studies, these may have been affected by external factors, such as changes in the guidelines. Therefore, we aimed to construct a predictive model and clarify the status of gastric ESD at our local community hospital before and after the publication of the supplementary guidelines to examine the risk factors for bleeding after gastric ESD.

## METHODS

### Study design

This was a retrospective study of gastric ESD cases registered in our hospital database. The authors conducted this study in accordance with the Declaration of Helsinki and obtained approval from the hospital's ethical review committee. Informed consent was obtained from all patients and their families.

### Description of setting

As a core hospital in our region, we targeted local residents for ESD without distinction. Approximately 100–150 gastric ESDs per year were performed by multiple gastroenterologists; proficiency varied from trainees to experts. Approximately 20 physicians were involved in this study.

The patients were admitted to the hospital one day prior to the procedure. ESD was performed using the Olympus‐Medical (Tokyo, Japan) Dual knife, Hook knife, and IT knife, as per the surgeon's preference. Sodium hyaluronate and saline solution were used for submucosal injection, and VIO 300D (ERBE, Germany) was used to generate high frequencies. Intraoperative anesthesia was administered with small doses of midazolam or flunitrazepam as a sedative and pethidine hydrochloride as the analgesic. Hemostasis of the ulcer base after ESD was achieved through cauterization using hemostatic forceps or the clip method; polyglycolic acid sheets were occasionally used to cover or close the ulcer base when using the clip method. Patients were administered 20 mg omeprazole twice daily as they were fasting on the day of treatment and the following day. Subsequently, omeprazole was replaced with 30 mg oral lansoprazole, 20 mg esomeprazole, or 20 mg vonoprazan fumarate before meals. Some surgeons performed second‐look endoscopy the day after treatment. Barring complications, most patients were discharged within 5–6 days postoperatively.

### Patient selection

We evaluated all cases of ESD performed at our hospital between May 2003 and July 2021 that were registered in the database. We followed up patients in the usual outpatient setting for ≥1 year, barring special circumstances. Patients with submucosal tumors, hyperplastic polyps, non‐carcinomas or non‐adenomas, or missing required data were excluded.

Cases were divided into two groups: those who underwent ESD before and those who underwent ESD after July 2017, the time of publication of the supplemental guidelines. If multiple lesions were resected en bloc, they were considered a single case. Anticoagulants were defined as warfarin and DOACs (dabigatran, rivaroxaban, apixaban, and edoxaban).

### Study variables

The variables in this study included patient characteristics (age and sex), medications at the time of ESD (types of anticoagulants, antiplatelets, and steroids), comorbidities at the time of ESD (atrial fibrillation, valvular disease, deep vein thrombosis, diabetes mellitus, hemodialysis, and liver cirrhosis), lesions (site, resection specimen size, procedure time, macroscopic type, ulcer, histology, and depth of invasion), and patient outcomes (curability of ESD, intraoperative and postoperative perforation, postoperative bleeding, death, and thromboembolism associated with withdrawal of anticoagulants). Antiplatelet agents included aspirin, cilostazol, and P2Y12 receptor antagonists. Anticoagulants and antiplatelet agents were collectively referred to as antithrombotic agents. Withdrawal and resumption of antithrombotic drugs were based on the guidelines and decisions of the attending physicians. However, a few patients discontinued the drugs independently or extended the withdrawal period at the discretion of their physicians. Post‐ESD bleeding was defined as hematemesis or hemoptysis and a decrease in post‐ESD peripheral blood hemoglobin level by at least 2.0 g/dl.

### Statistical analysis

R ver. 3.6.3 was used for statistical analysis (R Foundation for Statistical Computing, Vienna, Austria). The *t*‐test, Wilcoxon test, and chi‐square test were used to compare the two groups. Risk factors were evaluated using univariate analysis. The data of the pre‐publication cohort were used to predict bleeding using a logistic regression model. Variables that were significant in the univariate analysis and those identified as clinically significant in previous studies were included in the multivariate regression. Receiver‐operating characteristic (ROC) curves were created to evaluate the goodness of fit. Bootstrap was used to evaluate the internal validity. The prediction model created for the pre‐publication cohort was applied to the data of the post‐publication cohort to evaluate the external validity. The Kaplan–Meier curve was used to plot postoperative bleeding up to 14 days after ESD. Statistical significance was set at *p* < 0.05.

## RESULTS

**FIGURE 1:**
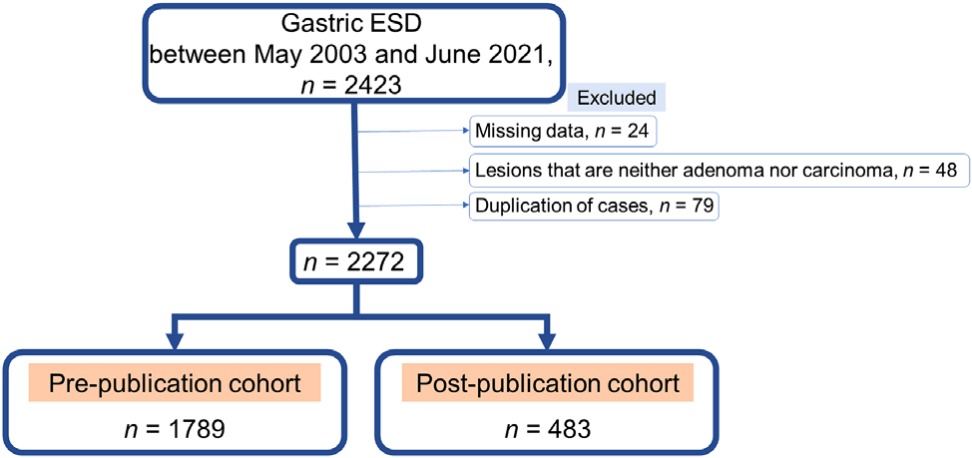
Allocation of endoscopic submucosal dissection (ESD) cases in our hospital into two groups. Of the 2423 cases registered in the database, 2272 were selected after excluding those with incomplete data. The pre‐publication cohort included 1789 cases of ESD performed before the publication of the supplementary guidelines, and the post‐publication cohort included 483 cases of ESD performed after the publication of the supplementary guidelines.

Our database included 2423 gastric ESD cases between May 2003 and June 2021. We limited gastric tumors to early carcinomas and adenomas and excluded other lesions and those with incomplete data and finally included 2272 cases. We assigned 1789 cases before and 483 cases after the publication of the supplemental guidelines to the pre‐ and post‐publication cohorts, respectively (Figure 1).

### Comparisons of patients, tumors, and outcomes

Comparisons of patient characteristics between the groups are summarized in Table 1a. There was an increase in the proportion of patients administered DOACs and a decrease in that administered warfarin. The proportion of patients with heparin replacement decreased, but there was no change in the incidences of underlying conditions. A comparison of gastric tumors between the groups is summarized in Table 1b; there was an increase in the percentage of lesions in the M region, decreased resection specimen size, and reduced procedure time. There was also an increase in the proportion of undifferentiated carcinomas. A comparison of outcomes between the groups is presented in Table 1c. Although the perforation rate decreased, there was no change in the incidence of post‐ESD bleeding. The overall post‐ESD bleeding rate was 2.68% (61/2272). Two cases of thromboembolism associated with withdrawal of anticoagulant agents accounted for 1.28% (2/152) of patients administered anticoagulants.

**TABLE 1a:** Comparison of patient characteristics between the pre and post‐publication cohorts

	**Pre‐publication cohort**	**Post‐publication cohort**	
	***n* = 1789**	***n* = 483**	***p***
Age, years (mean ± SD)	72.16 ± 8.39	73.06 ± 8.48	0.037
Male, *n* (%)	1351 (75.5)	366 (75.8)	0.954
Taking antithrombotics, *n* (%)			<0.001
None	1465 (81.9)	380 (78.7)	
Antiplatelet only	212 (11.9)	63 (13.0)	
Warfarin only	56 (3.1)	7 (1.4)	
Warfarin + antiplatelet	34 (1.9)	0 (0.0)	
DOAC only	19 (1.1)	28 (5.8)	
DOAC + antiplatelet	3 (0.2)	5 (1.0)	
Use of warfarin, *n* (%)	90 (5.0)	7 (1.4)	0.001
Use of DOAC, *n* (%)	22 (1.2)	33 (6.8)	<0.001
Heparin replacement, *n* (%)	72 (4.0)	6 (1.2)	0.005
AF, *n* (%)	101 (5.6)	37 (7.7)	0.124
Valvular disease, *n* (%)	7 (0.4)	2 (0.4)	1.000
DVT, *n* (%)	7 (0.4)	2 (0.4)	1.000
DM, *n* (%)	231 (12.9)	73 (15.1)	0.236
HD, *n* (%)	24 (1.3)	9 (1.9)	0.525
LC, *n* (%)	33 (1.8)	6 (1.2)	0.480
Use of steroid, *n* (%)	46 (2.6)	20 (4.1)	0.095

Abbreviations: AF, atrial fibrillation; DOAC, direct oral anticoagulants; DM, diabetes mellitus; DVT, deep vein thrombosis; HD, hemodialysis; LC, liver cirrhosis; SD, standard deviation.

**TABLE 1b:** Comparison of gastric lesions between the pre and post‐publication cohorts

	**Pre‐publication cohort**	**Post‐publication cohort**	
	***n* = 1789**	***n* = 483**	***p***
Location, *n* (%)			0.046
Upper	298 (16.7)	72 (14.9)	
Middle	503 (28.1)	165 (34.2)	
Lower	971 (54.3)	239 (49.5)	
Other	17 (1.0)	7 (1.4)	
Specimen size, mm (mean ± SD)	37.87 ± 14.77	35.29 ± 15.52	0.001
Procedure time, min (mean ± SD)	83.69 ± 77.55	71.47 ± 64.49	0.002
Depressed type, *n* (%)	807 (45.1)	229 (47.4)	0.395
Ulceration, *n* (%)	113 (6.3)	23 (4.8)	0.242
Undifferentiated, *n* (%)	26 (1.5)	25 (5.2)	<0.001
SM invasion, *n* (%)	174 (9.7)	49 (10.1)	0.851

Abbreviations: SD, standard deviation; SM, submucosa.

**TABLE 1c:** Comparison of outcomes between the pre and post‐publication cohorts

	**Pre‐publication cohort**	**Post‐publication cohort**	
	***n* = 1789**	***n* = 483**	***p***
Non‐curative resection, *n* (%)	198 (11.1)	53 (11.0)	1.000
Perforation, *n* (%)	66 (3.7)	3 (0.6)	<0.001
Bleeding, *n* (%)	50 (2.8)	11 (2.3)	0.641
Death, *n* (%)	3 (0.2)	0 (0.0)	0.846
Thromboembolism associated with withdrawal of anticoagulants, *n* (%)	2 (0.1)	0 (0.0)	1.000

### Risk factors for post‐ESD bleeding

Bleeding and non‐bleeding cases were compared in the pre‐ and post‐publication cohorts, and risk factors were examined in the univariate analysis (Table 2). Patients undergoing heparin replacement or hemodialysis had a higher risk for post‐ESD bleeding. For anticoagulants, 18.6% of all warfarin‐treated patients experienced bleeding, with a significant difference in the proportion experiencing bleeding in the pre‐publication cohort. For DOACs, there were fewer cases of bleeding, but a significant difference in the proportion of patients who experienced bleeding in the post‐publication cohort. However, there were no clear differences between types of DOAC. For lesion‐related factors, we found that resection specimen size may be a risk factor for post‐ESD bleeding.

**TABLE 2:** Comparison of clinicopathological features between bleeding and non‐bleeding cases in the pre and post‐publication cohorts

	**Pre‐publication cohort**			**Post‐publication cohort**		
	**No bleeding**	**Bleeding**		**No bleeding**	**Bleeding**	
	***n* = 1739**	***n* = 50**	***p***	***n* = 472**	***n* = 11**	***p***
Age, years (median)	73	75.5	0.170	74	75	0.913
Male, *n* (%)	1308 (96.8)	43 (3.2)	0.114	357 (97.5)	9 (2.5)	0.907
Heparin replacement, *n* (%)	55 (76.4)	17 (23.6)	<0.001	6 (100.0)	0 (0.0)	1.000
AF, *n* (%)	87 (86.1)	14 (13.9)	<0.001	34 (91.9)	3 (8.1)	0.057
Valvular disease, *n* (%)	5 (71.4)	2 (28.6)	0.003	2 (100.0)	0 (0.0)	1.000
DVT, *n* (%)	7 (100.0)	0 (0.0)	1.000	2 (100.0)	0 (0.0)	1.000
DM, *n* (%)	216 (93.5)	15 (6.5)	0.001	69 (94.5)	4 (5.5)	0.118
HD, *n* (%)	19 (79.2)	5 (20.8)	<0.001	6 (66.7)	3 (33.3)	<0.001
LC, *n* (%)	32 (97.0)	1 (3.0)	1.000	6 (100.0)	0 (0.0)	1.000
Use of steroid, *n* (%)	46 (100.0)	0 (0.0)	0.476	18 (90.0)	2 (10.0)	0.110
Anticoagulants, *n* (%)	93 (83.0)	19 (17.0)	<0.001	37 (92.5)	3 (7.5)	0.079
Taking warfarin, *n* (%)	72 (80.0)	18 (20.0)	<0.001	7 (100.0)	0 (0.0)	1.000
Warfarin only, *n* (%)	50 (89.3)	6 (10.7)	0.001	7 (100.0)	0 (0.0)	1.000
Warfarin + Antiplatelets, *n* (%)	22 (64.7)	12 (35.3)	<0.001	0 (0.0)	0 (0.0)	NA
Taking DOAC, *n* (%)	21 (95.5)	1 (4.5)	1.000	30 (90.9)	3 (9.1)	0.035
DOAC only, *n* (%)	18 (94.7)	1 (5.3)	1.000	26 (92.9)	2 (7.1)	0.260
DOAC + Antiplatelets, *n* (%)	3 (100.0)	0 (0.0)	1.000	4 (80.0)	1 (20.0)	0.245
Antiplatelets, *n* (%)	228 (91.6)	21 (8.4)	<0.001	64 (94.1)	4 (5.9)	0.087
Antiplatelets only, *n* (%)	203 (95.8)	9 (4.2)	0.253	60 (95.2)	3 (4.8)	0.335
Thienopyridine, *n* (%)	48 (94.1)	3 (5.9)	0.354	20 (90.9)	2 (9.1)	0.144
Anticoagulants + Antiplatelets, *n* (%)	25 (67.6)	12 (32.4)	<0.001	4 (80.0)	1 (20.0)	0.245
Number of Antithrombotics						
1 type, *n* (%)	244 (93.8)	16 (6.2)	0.001	85 (94.4)	5 (5.6)	0.055
2 or more, *n* (%)	52 (81.3)	12 (18.8)	<0.001	12 (92.3)	1 (7.7)	0.701
Location, *n* (%)			0.401			0.315
Upper	290 (97.3)	8 (2.7)		70 (97.2)	2 (2.8)	
Middle	484 (96.2)	19 (3.8)		164 (99.4)	1 (0.6)	
Lower	948 (97.6)	23 (2.4)		231 (96.7)	8 (3.3)	
Other	17 (100.0)	0 (0.0)		7 (100.0)	0 (0.0)	
Specimen size, mm, median	35.0	45.0	<0.001	32.0	38.0	0.141
Procedure time, min, median	65.0	77.5	0.162	50.0	60.0	0.876
Depressed type, *n* (%)	786 (97.4)	21 (2.6)	0.761	225 (98.3)	4 (1.7)	0.662
Ulceration, *n* (%)	109 (96.5)	4 (3.5)	0.840	23 (100.0)	0 (0.0)	0.973
Undifferentiated, *n* (%)	26 (100.0)	0 (0.0)	0.786	25 (100.0)	0 (0.0)	0.924
SM invasion, *n* (%)	166 (95.4)	8 (4.6)	0.202	48 (98.0)	1 (2.0)	1.000

Abbreviations: AF, atrial fibrillation; DM, diabetes mellitus; DOAC, direct oral anticoagulant; DVT, deep vein thrombosis; HD, hemodialysis; LC, liver cirrhosis; SM, submucosa.

### Predictive model for post‐ESD bleeding

In creating the predictive model in the pre‐publication cohort, we included known risk factors, such as antithrombotic agents, heparin replacement, and hemodialysis as explanatory variables when performing logistic regression analysis. Additionally, we added resection specimen size, which was a significant risk factor in the univariate analysis. We added the resection time as the last variable because it was expected to have a strong influence on post‐ESD bleeding. The cutoff values for resection specimen size and procedure time were 38 mm and 139 min, respectively, based on the ROC analysis. Five variables were incorporated into the model, within one‐tenth of the number of bleeding events, and we considered overfitting acceptable. The products of all the variables were fed into the model, and no interaction was found for any combination. The coefficient of variance magnification was <10 for all variables.

**FIGURE 2:**
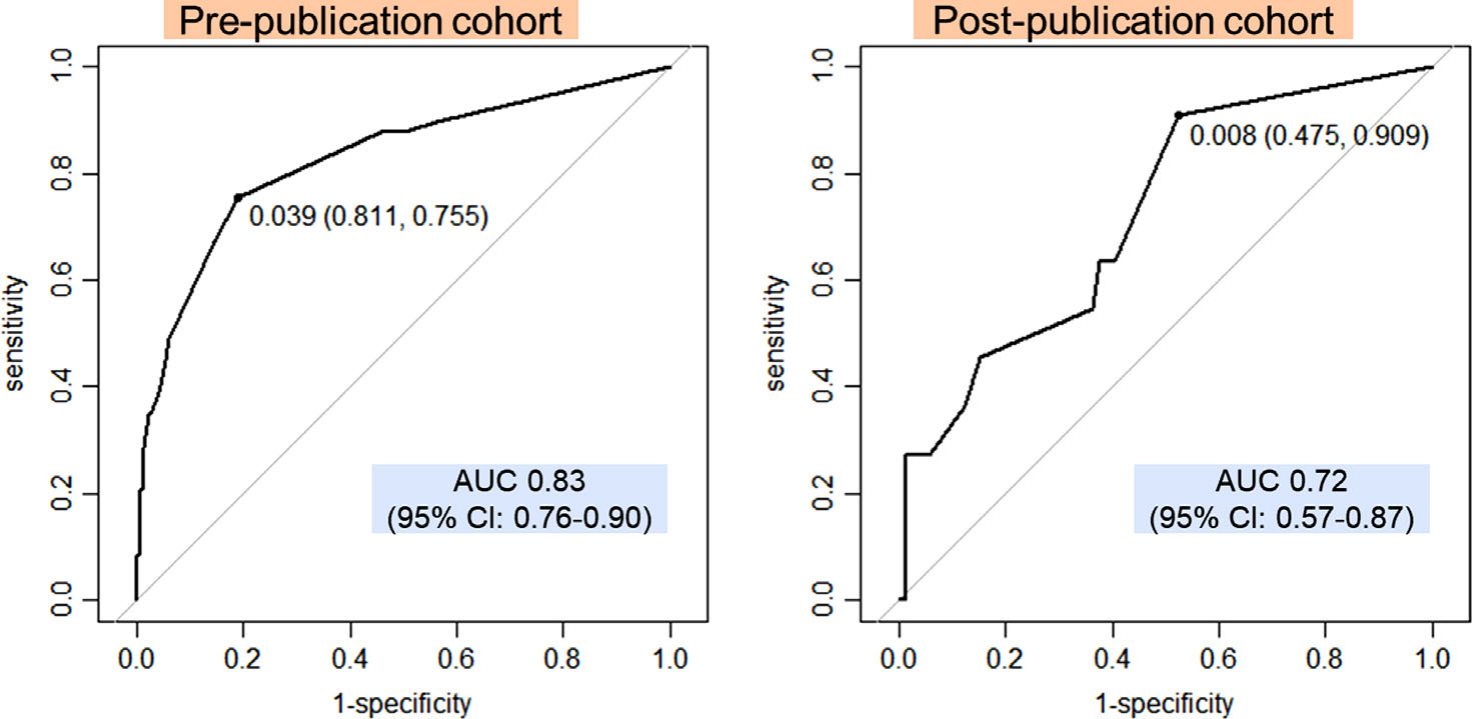
Receiver‐operating characteristic curve for the prediction model. The area under the curve (AUC) was 0.83 (95% confidence interval: 0.76–0.90) in the pre‐publication cohort and 0.72 (95% confidence interval: 0.57–0.87) in the post‐publication cohort.

The ROC curve was used to evaluate the goodness of fit of the model, and the C‐statistic was 0.83 (95% confidence interval [CI] 0.76–0.90) (Figure 2). Also, the Hosmer–Lemeshow test resulted in *p* = 0.984. When Bootstrap (*R* = 1000) was conducted to assess the internal validity, the mean C‐statistic was 0.83 (95% CI 0.76–0.89). When the model created in the pre‐publication cohort was incorporated into the post‐publication cohort to assess the external validity, the C‐statistic was 0.72 (95% CI 0.57–0.87).

The BEST‐J score,^13^ a predictive model with 10 variables, had an area under the ROC curve (area under the curve [AUC]) of 0.73 (95% CI 0.53–0.93) when applied to the post‐publication cohort of this study. We used DeLong's test to compare the AUC of the model in this study and the AUC based on the BEST‐J score, and found no significant difference (*p* = 0.911).

In the final model (Table 3), the risk of bleeding was quantified based on the partial regression coefficients for each variable. We set 1 point for one antithrombotic medication, 2 for ≥2 antithrombotic medications, 3 for heparin replacement and hemodialysis, 2 for resection specimen size ≥38 mm, and 1 for procedure time ≥139 min. We classified the total points of quantified bleeding risk into three groups: low‐risk (0–3 points: 1.6–4.0), intermediate‐risk (4–5 points: 8.4–13.1), and high‐risk (6–8 points: 21.2–34.8), with reference to the positive likelihood ratio.

**TABLE 3:** Logistic regression analysis of predictive factors for post‐ESD bleeding in the pre‐publication cohort

	**Odds rate**	**95% CI**	***p*‐value**	**B**	**Risk points** ^a^
Number of antithrombotics					
One type	2.22	0.96–4.81	0.050	0.799	1
Two or more	3.87	1.25–10.91	0.014	1.353	2
Heparin replacement	8.24	3.19–21.85	<0.001	2.059	3
HD	8.19	2.25–25.42	<0.001	2.099	3
Specimen size ≥38 mm	3.88	1.95–8.27	<0.001	1.362	2
Procedure time ≥139 min	2.03	0.97–4.07	0.051	0.715	1

B, partial regression coefficient; HD, hemodialysis

^a^ The risk of bleeding was quantified in risk points based on the partial regression coefficient of each variable.

Distributions of the risk score and classification in the pre‐publication cohort are summarized in Table 4. The bleeding rates at low, intermediate, and high risks were 1.6%, 10.3%, and 38.9%, respectively.

**TABLE 4:** Distribution of risk scores and risk classification for post‐ESD bleeding in the pre‐publication cohort

**Risk score**				**Risk classification**			
**Total points**	**Patients (*n* = 1789)**	**Bleeding (*n* = 50)**	**Rate of bleeding (%)**	**Risk categories**	**Patients (*n* = 1789)**	**Bleeding (*n* = 50)**	**Rate of bleeding (%)**
0	767	5	0.7	Low	1665	26	1.6
1	154	1	0.6				
2	502	6	1.2				
3	242	14	5.8				
4	62	6	9.7	Intermediate	87	9	10.3
5	25	3	12.0				
6	13	4	30.8	High	36	14	38.9
7	20	8	40.0				
8	3	2	66.7				
10	1	0	0.0				

**FIGURE 3:**
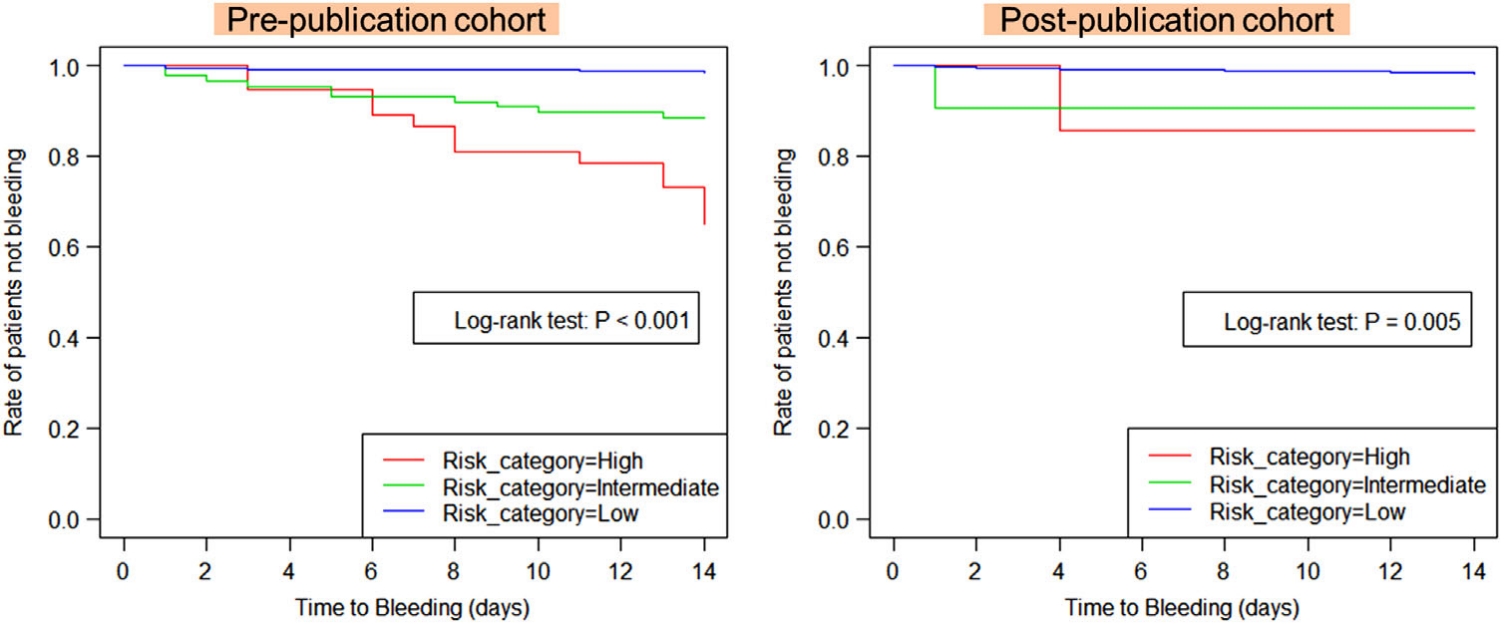
Relationship between each risk category and postoperative bleeding up to 14 days after endoscopic submucosal dissection. The Kaplan–Meier curve demonstrated a statistically significant difference in both the pre‐ and post‐publication cohorts, with a higher bleeding rate in the high‐risk group.

Similarly, the distributions of risk scores and classification in the post‐publication cohort are presented in Table 5. The bleeding rates were 0.9%, 9.5%, and 14.3% in the low‐risk, intermediate‐risk, and high‐risk groups, respectively. The bleeding rate in the high‐risk group was lower than that in the pre‐publication cohort. Finally, the time to bleeding curve for each risk category is shown in Figure 3; there was a significant difference in the incidence of postoperative bleeding up to 14 days after ESD in both the pre‐ and post‐publication cohorts.

**TABLE 5:** Distribution of risk scores and risk classification for post‐ESD bleeding in the post‐publication cohort

**Risk score**				**Risk classification**			
**Total points**	**Patients (*n* = 483)**	**Bleeding (*n* = 11)**	**Rate of bleeding (%)**	**Risk categories**	**Patients (*n* = 483)**	**Bleeding (*n* = 11)**	**Rate of bleeding (%)**
0	225	1	0.4	Low	632	6	0.9
1	74	3	1.4				
2	108	2	1.9				
3	48	2	4.2				
4	16	0	0.0	Intermediate	21	2	9.5
5	5	2	40.0				
6	7	1	14.3	High	7	1	14.3

## DISCUSSION

### Summary of results

In this study, we clarified the changes in gastric ESD before and after the publication of the supplementary guidelines. We also identified risk factors for post‐ESD bleeding and developed a predictive model. Despite a decrease in the number of patients administered warfarin and an increase in those administered DOACs after the publication of the supplementary guidelines, there was no significant difference in the rate of bleeding after gastric ESD. Furthermore, the type of DOAC was not an independent risk factor for post‐ESD bleeding.

### Interpretation of results

The shortened procedure time and reduced resection specimen size may be caused by improvements in the ESD techniques. The increase in the number of lesions in the M region and in the proportion of undifferentiated carcinoma may be due to improvements in endoscopic performance and image quality.

### Comparison with previous studies

Unlike previous reports,^10^ the type of DOAC was not an independent risk factor for post‐ESD bleeding in this study. We speculate that this was due to an insufficient number of patients and fewer bleeding events (apixaban, *n* = 2; rivaroxaban and edoxaban, *n* = 1 each). The definition of bleeding in this study was based on previous reports,^14^ and although slightly lower, the bleeding rate in this study was similar to those in previous reports. However, the resection specimen size was a significant risk factor for post‐ESD bleeding in this study, as in a previous study.^15^ One previous large study^13^ that also developed a predictive model for bleeding after ESD limited the cases to those with early gastric cancer, while our study included those with early gastric cancer and adenoma. Additionally, our study included cases treated after the publication of the supplemental guidelines, unlike previous studies.

### Clinical implications

Two cases of thromboembolism associated with anticoagulant withdrawal were observed in this study and involved warfarin replacement with heparin. The supplemental guidelines recommend endoscopic treatment with continuous warfarin therapy if the prothrombin time‐international normalized ratio is within the therapeutic range or a temporary change to DOAC for non‐valvular atrial fibrillation. Both cases were noted before the publication of the supplemental guidelines. Therefore, the supplement recommending short‐term withdrawal or continuation of antithrombotic agents may prevent thromboembolism without increasing the postoperative bleeding rate.

Only five variables were incorporated into the prediction model because of the number of events in the pre‐publication group. The following were significant: “two or more antithrombotic medications,” “heparin replacement,” “hemodialysis,” and “specimen size ≥38 mm.” All variables are known or expected to contribute to postoperative bleeding. “Two or more antithrombotic medications,” “heparin replacement,” “hemodialysis,” and “large specimen size” are reported risk factors for postoperative bleeding.^9^, ^10^, ^15^, ^16^ Although there are no reports of “procedure time” as a risk factor, we included it because it is likely to be influenced by a combination of the surgeon's skill, surgical difficulty, and frequency of intraoperative bleeding and hemostasis.

Regarding the Kaplan–Meier curve, the high‐risk group was clearly more likely to bleed within 14 days after the endoscopic procedure. Therefore, additional endoscopic observation should be considered not only immediately after but also during the 1–2 weeks after the procedure.

### Limitations and strengths

The reduction in the operative time before and after the publication of the guidelines suggests that ESD techniques may have improved over time, possibly introducing bias. Furthermore, this study was limited by its single‐center, retrospective nature. Compared with previous multicenter studies^15^ that developed prediction models for post‐ESD bleeding, fewer cases were included in this study; therefore, only five variables were included in the model. However, the performance of the model was good, with an AUC of 0.83, better than that in previous studies. Even after the publication of the supplementary guidelines, the model's accuracy remained good, with an AUC of 0.72. Generally, the accuracy of risk models declines as the environment changes over time.^17^ The model in this study functioned effectively even when the time axis was different and clinical practice was supposed to be different due to changes in the guidelines, thus indicating its robustness.

To the best of our knowledge, this is the first study to compare the results of a predictive model for post‐ESD bleeding before and after the publication of these supplementary guidelines. In other words, this study is the first to show that a prediction model based on pre‐publication data can be applied to post‐publication data and that the AUC for post‐publication data can be as good as 0.72. Furthermore, this study suggests that the BEST‐J score of Hatta et al.^13^ may also be applicable to daily practice. We conduct our daily practice in accordance with the supplemental guidelines and believe that this is of sufficient clinical significance. On the other hand, the predictive model in this study produced comparable results with fewer variables than the BEST‐J score. Although this is another strength of this study, it is necessary to examine the generalizability and inter‐institutional differences using multicenter studies in the future.

## CONCLUSION

There were changes in patient characteristics and clinical practice regarding ESD after the publication of the supplementary guidelines. Nevertheless, we developed a predictive model for post‐ESD bleeding that maintained accuracy over time. In the future, we would like to introduce the developed model into clinical practice and conduct interventional studies, such as the incorporation of prophylactic hemostasis in high‐risk patients and active endoscopic observation the day after ESD.

## CONFLICT OF INTEREST

The authors declare no conflict of interest.

## FUNDING INFORMATION

None.
